# Wanted: Standards for FAIR taxonomic concept representations and relationships

**DOI:** 10.3897/biss.5.75587

**Published:** 2021-09-23

**Authors:** Beckett Sterner, Nathan Upham, Prashant Gupta, Caleb Powell, Nico M Franz

**Affiliations:** ‡ Arizona State University, Tempe, United States of America

**Keywords:** FAIR Principles, open data, taxonomic intelligence

## Abstract

Making the most of biodiversity data requires linking observations of biological species from multiple sources both efficiently and accurately (Bisby 2000, Franz et al. 2016). Aggregating occurrence records using taxonomic names and synonyms is computationally efficient but known to experience significant limitations on accuracy when the assumption of one-to-one relationships between names and biological entities breaks down (Remsen 2016, Franz and Sterner 2018). Taxonomic treatments and checklists provide authoritative information about the correct usage of names for species, including operational representations of the meanings of those names in the form of range maps, reference genetic sequences, or diagnostic traits. They increasingly provide taxonomic intelligence in the form of precise description of the semantic relationships between different published names in the literature. Making this authoritative information Findable, Accessible, Interoperable, and Reusable (FAIR; Wilkinson et al. 2016) would be a transformative advance for biodiversity data sharing and help drive adoption and novel extensions of existing standards such as the Taxonomic Concept Schema and the OpenBiodiv Ontology (Kennedy et al. 2006, Senderov et al. 2018). We call for the greater, global Biodiversity Information Standards (TDWG) and taxonomy community to commit to *extending and expanding* on how FAIR applies to biodiversity data and include practical targets and criteria for the publication and digitization of taxonomic concept representations and alignments in taxonomic treatments, checklists, and backbones.

As a motivating case, consider the abundantly sampled North American deer mouse— *Peromyscus maniculatus* (Wagner 1845)—which was recently split from one continental species into five more narrowly defined forms, so that the name *P. maniculatus* is now only applied east of the Mississippi River (Bradley et al. 2019, Greenbaum et al. 2019). That single change instantly rendered ambiguous ~7% of North American mammal records in the Global Biodiversity Information Facility (n=242,663, downloaded 2021-06-04; GBIF.org 2021) and ⅓ of all National Ecological Observatory Network (NEON) small mammal samples (n=10,256, downloaded 2021-06-27). While this type of ambiguity is common in name-based databases when species are split, the example of *P. maniculatus* is particularly striking for its impact upon biological questions ranging from hantavirus surveillance in North America to studies of climate change impacts upon rodent life-history traits. Of special relevance to NEON sampling is recent evidence suggesting deer mice potentially transmit SARS-CoV-2 (Griffin et al. 2021).

Automating the updating of occurrence records in such cases and others will require operational representations of taxonomic concepts—e.g., range maps, reference sequences, and diagnostic traits—that are FAIR *in addition to* taxonomic concept alignment information (Franz and Peet 2009). Despite steady progress, it remains difficult to find, access, and reuse authoritative information about how to apply taxonomic names even when it is already digitized. It can also be difficult to tell without manual inspection whether similar types of concept representations derived from multiple sources, such as range maps or reference sequences selected from different research articles or checklists, are in fact interoperable for a particular application. The issue is therefore different from important ongoing efforts to digitize trait information in species circumscriptions, for example, and focuses on how already digitized knowledge can best be packaged to inform human experts and artifical intelligence applications (Sterner and Franz 2017). We therefore propose developing community guidelines and criteria for ***FAIR taxonomic concept representations*** as “semantic artefacts” of general relevance to linked open data and life sciences research (Le Franc et al. 2020).
